# Conditional and unconditional genome-wide association study reveal complicate genetic architecture of human body weight and impacts of smoking

**DOI:** 10.1038/s41598-020-68935-x

**Published:** 2020-07-22

**Authors:** Ting Xu, Md. Mamun Monir, Xiang-Yang Lou, Haiming Xu, Jun Zhu

**Affiliations:** 1 Department of Mathematics, Zhejiang University, Hangzhou, 310058 China; 2 Institute of Bioinformatics, Zhejiang University, Hangzhou, 310058 China; 3 Department of Biostatistics, University of Florida, Gainesville, FL 32611 USA

**Keywords:** Quantitative trait, Genetic association study

## Abstract

To reveal the impacts of smoking on genetic architecture of human body weight, we conducted a genome-wide association study on 5,336 subjects in four ethnic populations from MESA (The Multi-Ethnic Study of Atherosclerosis) data. A full genetic model was applied to association mapping for analyzing genetic effects of additive, dominance, epistasis, and their ethnicity-specific effects. Both the unconditional model (base) and conditional model including smoking as a cofactor were investigated. There were 10 SNPs involved in 96 significant genetic effects detected by the base model, which accounted for a high heritability (61.78%). Gene ontology analysis revealed that a number of genetic factors are related to the metabolic pathway of benzopyrene, a main compound in cigarettes. Smoking may play important roles in genetic effects of dominance, dominance-related epistasis, and gene-ethnicity interactions on human body weight. Gene effect prediction shows that the genetic effects of smoking cessation on body weight vary from different populations.

## Introduction

Body weight is a typical complex trait in human. Classical genetic studies have shown that the heritability of obesity is about 40% to 70%^1–3^. Compared with BMI (body mass index) and body surface, body weight can be easily and accurately measured, and thus is convenient to use for scientific researches.

Over the past two decades, more than forty candidate genes associated with human body weight have been found. Among them, many genes (*FTO, MC4R, KCTD15, TMEM18, SH2B1, GNPDA2, MTCH2, NEGR1, TNNI3K, QPCTL, BDNF*) have been reported several times, and genes *FTO* and *MC4R* were ones of the mostly confirmed by many studies^1,4,5^. The *FTO* gene is the first weight-related gene to be discovered with specific function and metabolic pathways being explained clearly by functional and molecular biology^6^. Nevertheless, the estimated heritability of these weight-related genes is still low. Even if we add all the heritability of above-mentioned genes together, the result is less than 10%^2,3^, and majority of heritability is missing, coined as missing heritability. Epistasis and gene-environment (e.g., life style factors) interactions are considered as one of the primary reasons for missing heritability.

Acquired behaviors may have influences on body weight by genetic effects of weight-related genes, such as *FTO*^7^. Smoking is a series social problem and has direct influences on human body weight. A consensus opinion is that the average weight of smokers is lower than nonsmokers, and quitting smoking will lead to weight gain^8^. One article suggested that smoking may change the sensitization potential of body fat distribution^9^. Moreover, mother smoking during pregnancy is a possible factor leading to childhood obesity^10,11^.

Cracking genetic architecture of human body weight and identification of the genes related to weight is a major task. To reveal the genetic architecture of human body weight and the impact of smoking, we have conducted a genome-wide association study (GWAS) on body weight (WT) in a multi-ethnic genetic study by a model including additive effects, dominance effects, epistasis effects as well as their ethnicity interactions. For detecting impacts of smoking on genetic effects of human body weight, pack-years of cigarette smoking (SMK) was set as a covariant by conducting conditional association mapping^12^ in the conditional model (WT|SMK). For analyzing genetic models, estimating genetic effects, and improving test efficiency at reasonable computational costs, a mixed linear model approach was used^13^. The findings will provide useful information for tailored prevention and precision treatment.

## Materials and methods

### Study sample

The Multi-Ethnic Study of Atherosclerosis (MESA) is a study of the characteristics of cardiovascular disease and its physiological and social psychological risk factors^14^. Several thousand participants recruited form six US communities had physical examinations, and the genotype data was obtained from gene sequencing. 5,336 individuals passed the preliminary screening (repeat of two years with missing data, contained 9,984 observations). There are 38% of European–American (E-A population), 28% of African–American (A-A population), 22% of Hispanic–American (H-A population), 12% of Chinese–American (C-A population). The gender is basically evenly distributed in each ethnic population. The MESA dataset was obtained from the dbGaP (the database of Genotypes and Phenotypes) of US National Center for Biotechnology Information website (https://www.ncbi.nlm.nih.gov/gap, accession number: phs000209.v11.p3).

### Genotype and quality control

Before the formal study began, we performed quality control on the original genotype data. Single nucleotide polymorphism (SNP) with call rate less than 95% was deleted. Polymorphic at least in one ethnic group monomorphic SNPs in each ethnic group were deleted. Due to the allelic frequency differences between ethnic groups, no SNPs were filtered based on the minor allele frequency. The uniform heterozygosity distribution is limited to the range of 0–53%, and SNPs with heterozygosity larger than 53% were removed. And we also filtered the SNP with Hardy weinberg equilibrium (HWE) *P*-value larger than 1 × 10^–4^. Finally, 866,435 SNPs from 22 autosomes met the quality control standards and were used for our analysis.

### Phenotype and the cofactor

The age, gender, ethnicity, and other basic information including smoking habits of each study participant were assessed in the form of a set of self-reporting questionnaires. Physiological index like body weight and height was obtained by physical examinations, which were conducted twice, the second time was two years later than the first time. We took body weight (the variable name is “wtlb1”, the values were recorded in pounds) as the main object. Smoking (the variable name is “pkyrslc”, which means the average number of packets of cigarettes someone smoked per year) was chosen in this study as covariant.

### Statistical analysis

In this study, mixed linear model approaches were used for genome-wide association studies. The full model includes additive and dominance effects of singular SNPs (*a*, *d*), epistasis effects between pairs of SNPs (*aa*, *ad*, *da*, *dd*), and ethnicity-specific effects (*e*, *ae*, *de*, *aae*, *ade*, *dae*, *dde*). The phenotypic values of the *k-th* individual in the *h-th* ethnic population (${y}_{hk}$) can be expressed as

$$ \begin{aligned} y_{hk} & = \mu + c_{hk} + s_{hk} + \sum\limits_{i} {a_{i} u_{{A_{ik} }} } + \sum\limits_{i} {d_{i} u_{{D_{ik} }} } + \sum\limits_{i < j} {aa_{ij} u_{{AA_{ijk} }} } + \sum\limits_{i < j} {ad_{ij} u_{{AD_{ijk} }} } + \sum\limits_{i < j} {da_{ij} u_{{DA_{ijk} }} } + \sum\limits_{i < j} {dd_{ij} u_{{DD_{ijk} }} } \hfill \\ & \quad + e_{h} + \sum\limits_{i} {ae_{ih} u_{{AE_{ihk} }} } + \sum\limits_{i} {de_{ih} u_{{DE_{ihk} }} } + \sum\limits_{i < j} {aae_{ijh} u_{{AAE_{ijhk} }} } + \sum\limits_{i < j} {ade_{ijh} u_{{ADE_{ijhk} }} } + \sum\limits_{i < j} {dae_{ijh} u_{{DAE_{ijhk} }} } + \sum\limits_{i < j} {dde_{ijh} u_{{DDE_{ijhk} }} } + \varepsilon_{hk} \hfill \\ \end{aligned} $$

where $\mu$ is the population mean; $c_{hk}$ is the fixed cofactor effects; $s_{hk}$ is the fixed gender effects; $a_{i}$ is the additive effect of the *i-th* locus with coefficient $u_{{A_{ik} }}$; $d_{i}$ is the dominance effect of the *i-th* locus with coefficient $u_{{D_{ik} }}$; $aa_{ij}$, $ad_{ij}$, $da_{ij}$ and $dd_{ij}$ are the digenic epistasis effects with coefficients $u_{{AA_{ijk} }}$, $u_{{AD_{ijk} }}$, $u_{{DA_{ijk} }}$ and $u_{{DD_{ijk} }}$; $e_{h}$ is the effect of the *h*-*th* ethnic population (1 for E-A, 2 for C-A, 3 for A-A, 4 for H-A); $ae_{ih}$ is the additive × ethnicity interaction effect of the *i*-*th* locus in the *h*-*th* ethnic population with coefficient $u_{{AE_{ihk} }}$; $de_{ih}$ is the dominance × ethnicity interaction effect of the *i*-*th* locus in the *h*-*th* ethnic population with coefficient $u_{{DE_{ihk} }}$; $aae_{ijh}$, $ade_{ijh}$, $dae_{ijh}$ and $dde_{ijh}$ are the ethnicity-specific epistasis effects in the *h*-*th* ethnic population with coefficient $u_{{AAE_{ijhk} }}$, $u_{{ADE_{ijhk} }}$, $u_{{DAE_{ijhk} }}$ and $u_{{DDE_{ijhk} }}$; and $\varepsilon_{hk}$ is the residual effect of the *k-th* individual in the *h-th* ethnic population.

In our mixed linear model, we take ethnicity-specific effects into consideration, instead of detecting them separately in each ethnic population. The general effects (*a*, *d*, *aa*, *ad*, *da*, *dd*) are effects stably expressed in each ethnic population, while the ethnicity-specific effects are specifically expressed in a certain population.

Conditional genetic model is an effective method to reveal conditional effects excluding interference factors^15^. If we add smoking variables in the mixed linear model as cofactor, it could reveal net genetic effects after removing influence of smoking.

To reduce the computational burden in mixed model-based GWAS analysis, we first used GMDR (Generalized Multifactor Dimensionality Reduction)^16^ method to scan 866,435 SNPs of 9,984 observations in one dimension (single SNP), two dimensions (two SNPs epistasis), and three dimensions (three SNPs epistasis), and a set of 807 candidate SNPs were obtained. In the second step, a GPU parallel computing software ***QTXNetwork*** was used to run the mixed linear model on 807 candidate SNPs. The estimated genetic effects and the standard errors (*SE*) of detected SNPs were obtained by Monte Carlo Markov Chain method with 20,000 Gibbs sampler iterations. The experiment-wise *P* values (*P*_EW_-value) for controlling the experiment-wise type I error (*P*_EW_ < 0.05) were calculated by 2,000 permutation tests^17^.

### Bioinformatics analysis

We also search the detected genes for biological information through the text-mining search engine ***BiopubInfo***. ***BiopubInfo*** is a full-text biological literature search engine based on words and phrases developed by our laboratory. The literature data includes all abstract databases of PubMed and full-text databases of more than 2,500 magazines. In order to realize the identification of different biomedical concepts by ***BiopubInfo***, we sorted out the different expressions and synonyms of all concepts, and the hierarchical and subordinate relationships between concepts using 118 ontology databases including UMLS^18^, OLS^19^, BioOntology.org^20^ to build a database of biomedical concepts. In this research, we use ***BiopubInfo*** to search genetic networks between weight-related genes with chemicals, diseases, and gene-gene interactions.

### Estimation of genetic effects

As is mentioned above, mixed linear model can estimate genetic effects with their standard errors^12^. For a person of a certain ethnicity, we calculate the sum of the general effects and the ethnicity-specific effect of this ethnicity of all the weight-related SNPs to obtain the estimated value of his body weight. For simplicity and intuitively, we showed the estimation data, a 5,336 × 15 matrix, in the form of figure. We compared estimated genetic effects of base model (WT) to conditional model (WT|SMK), and revealed impacts of smoking on gene effects of body weight.

## Results

### Detected SNPs

**Figure 1:**
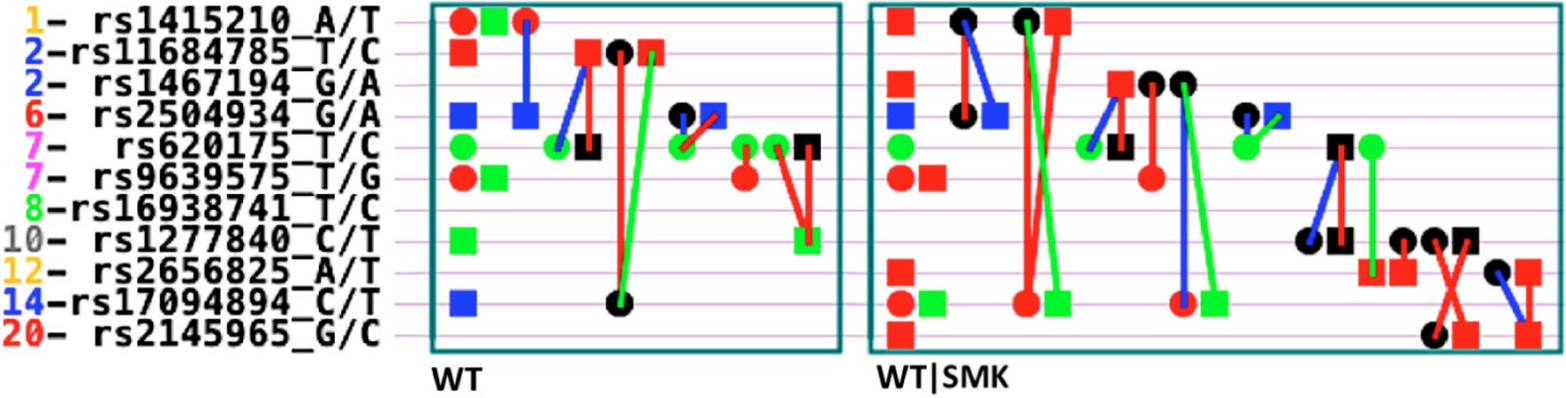
Genetic architecture of highly significant SNPs at the experiment-wise level (*P*_*EW*_ < 10^–5^) for body weight in two models. WT = the base model with no cofactor (left box). WT|SMK = the conditional model on smoking (right box). The left axis is the SNP IDs (Chromosome_SNP_Allele), and each line represents a SNP. The different shape symbols on each line represent the different effects of that SNP: Circle = additive effect; Square = dominance effect; Black color = only epistasis effects (not include single effects); Red color = general effects for four ethnic groups; Green color = ethnicity-specific effects; Blue color = both general and ethnicity-specific effects; Lines = epistasis effect of two SNPs, and the color of the lines have the same meaning as above.

Eleven SNPs were identified significantly by two models (seven SNPs in the base model WT and nine SNPs in the conditional model WT|SMK). Highly significant SNPs at the experiment-wise level (*P*_*EW*_ < 10^–5^) are presented in Fig. 1. We use different shapes (circle and square) and colors (green, red, blue and black) to show different kinds of genetic effects of SNPs.

The estimated genetic effects (represented by the various colors and shapes in Fig. 1) and heritabilities of two models are summarized in Tables 1, 2 and 3, revealing the genetic patterns and gene-ethnicity interactions on body weight.

**Table 1:** Estimated heritability of significant SNPs for body weight in two different models.

Model	$h_{A}^{2}$ (%)	$h_{D}^{2}$ (%)	$h_{I}^{2}$ (%)	$h_{AE}^{2}$ (%)	$h_{DE}^{2}$ (%)	$h_{IE}^{2}$ (%)	$h_{T}^{2}$ (%)
WT	1.63	4.29	9.82	1.53	18.30	26.21	61.78
WT|SMK	0.63	9.15	13.86	1.49	7.87	33.91	66.91

Model: WT = the base model with no cofactor. WT|SMK = the conditional model with smoking as a cofactor.

Heritability: $h_{A}^{2}$ = heritability of additive effects; $h_{D}^{2}$ = heritability of dominance effects; $h_{I}^{2}$ = heritability of epistasis effects including *AA*, *AD, DA, DD;* $h_{AE}^{2}$ = heritability of ethnicity-specific additive interaction effects; $h_{DE}^{2}$ = heritability of ethnicity-specific dominance effects; $h_{IE}^{2}$ = heritability of ethnicity-specific epistasis effects including *AAE*, *ADE, DAE, DDE;* $h_{T}^{2}$ = total heritability.

**Table 2:** Estimated genetic effects of highly significant loci for body weight in the base and conditional models.

Chr_SNP_Allele (major/minor)	Gene	Effect	Estimate	*SE*	–Log*P*_*EW*_	$h^{2}$ (%)	Smoke
1_rs1415210_A/T	*CRB1*	*a*	4.13	0.42	22.12	0.62	√
		*de*_*1*_	3.46	0.73	5.69	0.43	√
2_rs11684785_T/C	*TMEM163*	*d*	6.26	0.51	33.57	1.41	×
6_rs2504934_G/A	*SLC22A3*	*d*	4.22	0.63	10.81	0.64	×
		*de*_*1*_	– 7.59	0.86	17.98	2.09	×
		*de*_*3*_	22.27	1.52	47.43	17.93	×
		*de*_*4*_	– 13.67	1.14	32.31	6.76	×
7_rs620175_T/C	*SDK1*	*ae*_*2*_	– 5.26	0.91	8.05	1.00	√
		*ae*_*3*_	8.74	0.94	19.70	2.76	×
		*ae*_*4*_	– 5.68	0.85	10.73	1.17	×
7_rs9639575_T/G	*CREB5*	*a*	– 5.12	0.41	35.65	0.95	×
		*de*_*4*_	– 4.59	1.03	5.05	0.76	√
10_rs1277840_C/T	*CACNB2*	*de*_*3*_	– 5.11	0.96	7.05	0.95	√
14_rs17094894_C/T	175 kb 3′ of *VRK1*	*d*	6.94	0.92	13.19	1.74	√
		*de*_*4*_	– 10.54	1.37	13.84	4.02	×
1_rs1415210_A/T × 6_rs2504934_G/A	*CRB1* × *SLC22A3*	*ad*	–5.87	0.83	11.69	1.25	×
		*ade*_*1*_	5.23	1.18	5.03	0.99	√
		*ade*_*3*_	– 9.51	1.73	7.43	3.27	×
2_rs11684785_T/C × 7_rs620175_T/C	*TMEM163* × *SDK1*	*da*	– 4.02	0.62	10.03	0.58	×
		*dae*_*1*_	– 6.92	0.91	13.62	1.73	×
		*dd*	– 7.74	0.90	16.94	2.16	×
2_rs11684785_T/C × 14_rs17094894_C/T	*TMEM163* × 175 kb 3′ of *VRK1*	*aa*	4.56	0.45	23.39	0.75	×
		*dae*_*1*_	4.51	0.76	8.55	0.73	√
6_rs2504934_G/A × 7_rs620175_T/C	*SLC22A3* × *SDK1*	*aa*	– 6.05	0.46	38.26	1.32	×
		*aae*_*1*_	5.28	0.75	11.55	1.01	×
		*aae*_*2*_	– 4.17	0.92	5.26	0.63	×
		*aae*_*3*_	– 7.71	1.06	12.52	2.15	×
		*aae*_*4*_	7.81	1.06	12.70	2.20	×
		*da*	3.45	0.77	5.17	0.43	√
7_rs620175_T/C × 7_rs9639575_T/G	*SDK1* × *CREB5*	*aa*	2.43	0.48	6.32	0.21	√
7_rs620175_T/C × 10_rs1277840_C/T	*SDK1* × *CACNB2*	*ad*	3.03	0.62	6.00	0.33	√
		*dd*	6.46	0.81	14.92	1.51	×

Model: Smoke = model setting smoking as a cofactor. √: effect caused by smoking. ×: effect not affected by smoking.

Genetic effect: *a* = additive effect, *d* = dominance effect, *aa* = additive × additive epistasis effect, *ad* = additive × dominance epistasis effect, *da* = dominance × additive epistasis effect, *dd* = dominance × dominance epistasis effect, *ae*_*2*_ = C-A specific additive effect, *ae*_*3*_ = A-A specific additive effect, *ae*_*4*_ = H-A specific additive effect, *de*_*1*_ = E-A specific dominance effect, *de*_*3*_= A-A specific dominance effect, *de*_*4*_= H-A specific dominance effect, *aae*_*1*_ = E-A specific additive × additive epistasis effect, *aae*_*2*_ = C-A specific additive × additive epistasis effect, *aae*_*3*_ = A-A specific additive × additive epistasis effect, *aae*_*4*_ = H-A specific additive × additive epistasis effect, *ade*_*1*_ = E-A specific additive × dominance epistasis effect, *ade*_*3*_ = A-A specific additive × dominance epistasis effect, *dae*_*1*_ = E-A specific dominance × additive epistasis effect. − Log*P*_*EW*_ = minus log_10_(experiment-wise *P*-value). *h*^2^ = heritability.

**Table 3:** Estimated genetic effects of highly significant loci for body weight only in conditional model.

Chr_SNP_Allele (major/minor)	Gene	Effect	Estimate	*SE*	–LogP_*EW*_	*h*^2^%
1_rs1415210_A/T	*CRB1*	*d*	– 4.42	0.51	17.03	0.62
7_rs9639575_T/G	*CREB5*	*d*	2.85	0.54	6.78	0.26
12_rs2656825_A/T	*LOC101927616*	*d*	9.06	0.53	64.21	2.62
14_rs17094894_C/T	175 kb 3ʹ of *VRK1*	*a*	– 3.23	0.35	19.81	0.33
20_rs2145965_G/C	184 kb 5ʹ of *KIZ*	*d*	6.42	0.50	37.25	1.32
1_rs1415210_A/T × 6_rs2504934_G/A	*CRB1* × *SLC22A3*	*aa*	– 2.70	0.48	7.62	0.23
1_rs1415210_A/T × 14_rs17094894_C/T	*CRB1* × 175 kb 3ʹ of *VRK1*	*aa*	– 6.44	0.45	46.37	1.33
		*da*	3.12	0.55	7.80	0.31
		*ade*_*4*_	– 9.97	1.88	6.92	3.18
2_rs1467194_G/A × 7_rs9639575_T/G	*TMEM163* × *SDK1*	*aa*	– 3.51	0.53	10.53	0.39
2_rs1467194_G/A × 14_rs17094894_C/T	*TMEM163* × 175 kb 3ʹ of *VRK1*	*aae*_*1*_	3.45	0.67	6.50	0.38
		*ade*_*2*_	8.44	1.59	6.99	2.28
6_rs2504934_G/A × 7_rs620175_T/C	*SLC22A3* × *SDK1*	*dae*_*1*_	6.60	1.02	10.00	1.39
		*dae*_*3*_	– 11.76	2.06	7.92	4.42
		*dae*_*4*_	6.12	1.38	5.05	1.20
7_rs620175_T/C × 10_rs1277840_C/T	*SDK1* × *CACNB2*	*da*	– 3.95	0.81	5.96	0.50
		*dae*_*3*_	– 5.74	1.30	5.02	1.05
7_rs620175_T/C × 12_rs2656825_A/T	*SDK1* × *LOC 101927616*	*ade*_*1*_	– 4.53	0.98	5.44	0.66
		*ade*_*3*_	8.52	1.33	9.82	2.32
10_rs1277840_C/T × 12_rs2656825_A/T	*CACNB2* × *LOC101927616*	*ad*	3.42	0.73	5.54	0.37
10_rs1277840_C/T × 20_rs2145965_G/C	*CACNB2* × 184 kb 5ʹ of *KIZ*	*ad*	– 3.60	0.69	6.83	0.42
		*da*	3.06	0.67	5.33	0.30
12_rs2656825_A/T × 20_rs2145965_G/C	*LOC101927616* × 184 kb 5ʹ of *KIZ*	*ad*	– 3.17	0.63	6.23	0.32
		*dd*	– 7.77	0.80	21.57	1.93
		*ade*_*1*_	– 5.17	0.89	8.12	0.85

Genetic effect: *a* = additive effect, *d* = dominance effect, *aa* = additive × additive epistasis effect, *ad* = additive × dominance epistasis effect, *da* = dominance × additive epistasis effect, *dd* = dominance × dominance epistasis effect, *aae*_*1*_ = E-A specific additive × additive epistasis effect, *ade*_*1*_ = E-A specific additive × dominance epistasis effect, *ade*_*2*_ = C-A specific additive × dominance epistasis effect, *ade*_*3*_ = A-A specific additive × dominance epistasis effect, *ade*_*4*_ = H-A specific additive × dominance epistasis effect, *dae*_*1*_ = E-A specific dominance × additive epistasis effect, *dae*_*3*_ = A-A specific dominance × additive epistasis effect, *dae*_*4*_ = H-A specific dominance × additive epistasis effect. − Log*P*_*EW*_ = minus log_10_(experiment-wise *P*-value). *h*^2^ = heritability.

### Estimated heritability of different models

Estimated heritability of the base model (WT) and conditional model (WT|SMK) are presented in Table 1. The total heritability of human body weight in the base model is up to 61.78%, within the range of heritability for human body weight discussed in the previous studies (from 40 to 70%). The heritability is mostly contributed by ethnicity-specific dominance effects ($h_{DE}^{2} \overset{\wedge}=$ 18.30%) and ethnicity-specific epistasis effects ($h_{IE}^{2} \overset{\wedge}=$ 26.21%: $h_{AAE}^{2} \overset{\wedge}=$ 1.67%, $h_{ADE}^{2} \overset{\wedge}=$ 13.66%, $h_{DAE}^{2} \overset{\wedge}=$ 5.49%, $h_{DDE}^{2} \overset{\wedge}=$ 5.39%). SNP rs2504934 in the *SLC22A3* gene has the highest heritability ($h_{{de_{3} }}^{2} \overset{\wedge}=$ 17.93%) in A-A population, and it is the leading result of high heritability of ethnicity-specific dominance effects ($h_{DE}^{2} \overset{\wedge}=$ 18.30%). The C/C of rs11684785 × C/T of rs17094894 (*TMEM163* × near *VRK1*) has the highest epistasis heritability ($h_{{ade_{1} }}^{2} \overset{\wedge}=$ 20.61%) in E-A population, and it leads to high heritability of ethnicity-specific additive × dominance epistasis effect ($h_{ADE}^{2} \overset{\wedge}=$ 13.66%) with high significance (*P*_*EW*_ -value = 7.43 × 10^−5^).

The total heritability of conditional model on smoking ($h_{T}^{2} \overset{\wedge}=$ 66.91%) is higher than that of base model, mostly due to epistasis effects ($h_{I}^{2} \overset{\wedge}=$ 13.86%), and ethnicity-specific epistasis effects ($h_{IE}^{2} \overset{\wedge}=$ 33.91%). The highest heritability ($h_{{dae_{3} }}^{2} \overset{\wedge}=$ 4.42%) belongs to G/A of rs2504934 in the *SLC22A3* gene × T/T of rs620175 in the *SDK1* gene. This weight-decreasing epistasis was not detected in the base model, indicating that this epistasis is suppressed by smoking.

### Genetic effects of SNPs related to human body weight

There were 32 highly significant genetic effects (*P*_*EW*_-value < 1 × 10^−5^) of human body weight detected in the base model (Table 2). Among all the genetic effects, nearly half of them are ethnicity-specific effects, but C-A population has fewer (2/18) ethnicity-specific effects, suggesting that weight-related genes have minor specific effects in Chinese-American.

### Genetic effects of SNPs not affected by smoking

When removing impacts of smoking in the conditional model, we detected 13 negative effects and eight positive effects of body weight not affected by smoking (Table 2).

The homozygote genotype of rs9639575 has additive effect ($a \overset{\wedge}=$ – 5.12 for T/T and 5.12 for G/G), which reveals that major frequency homozygous (T/T) has negative additive effect, but minor frequency homozygous (G/G) has positive additive effect. The heterozygote genotype T/C of rs11684785 has a positive dominance effect ($d \overset{\wedge}=$ 6.26) and three negative epistasis effects with interaction to homozygote genotype T/T and heterozygote genotype T/C of rs620175 ($da \overset{\wedge}=$ – 4.02 for T/C × T/T, but 4.02 for T/C × C/C; $dae_{1} \overset{\wedge}=$ – 6.92 for T/C × T/T, but 6.92 for T/C × C/C. $dd \overset{\wedge}=$ – 7.74).

One dominance effect and three ethnicity-specific dominance effects of the heterozygote genotype G/A of rs2504934 will increase body weight by 4.22 lb, but decrease body weight by – 7.59 lb in E-A population and – 13.67 in H-A population, increase body weight by 22.27 lb in A-A population, respectively. Interestingly, this SNP not only has the largest positive effect but also the smallest negative effect among all genetic effects with highly significance ($de_{3} \overset{\wedge}=$ 22.27, *P*_*EW*_-value < 1 × 10^−47^; $de_{4} \overset{\wedge}=$ – 13.67, *P*_*EW*_-value < 1 × 10^−32^). This means SNPs can have totally different effects in different ethnic populations. The homozygote genotype G/G of this SNP also has five different epistasis effects with interaction to rs620175 in the *SDK1* gene (additive × additive effect and additive × additive epistasis effects in all four populations, increases body weight in E-A and H-A populations, decreases body weight in C-A and A-A population), indicating that this epistasis regulates body weight through different metabolic networks in different populations and has widespread effects in different populations. These results show that SNP rs2504934 in the *SLC22A3* gene has major effects on body weight, and it is not affected by smoking.

Genetic effects of rs620175 in the *SDK1* gene have large parts of total effects (11/21). In addition to single effects ($ae_{3} \overset{\wedge}=$ 8.74 for T/T but – 8.74 for C/C, $ae_{4} \overset{\wedge}=$ – 5.68 for T/T but 5.68 for C/C), SNP rs620175 also has many epistasis effects with interaction to three SNPs (rs11684785 in the *TMEM163* gene, rs2504934 in the *SLC22A3* gene, and rs1277840 in the *CACNB2* gene). Epistasis rs620175 × rs1277840 has positive dominance × dominance effect ($dd \overset{\wedge}=$ 6.46), while all the effect values of rs11684785 × rs620175 are negative ($da \overset{\wedge}=$ – 4.02 for T/C × T/T, $dd \overset{\wedge}=$ – 7.74, $dae_{1} \overset{\wedge}=$ – 6.92 for T/C × T/T).

### Genetic effects of SNPs caused by smoking

Eleven highly significant genetic effects were detected due to smoking (Table 2). All five single effects and ethnicity-specific effects in E-A population are positive, but ethnicity-specific effects in other three ethnic populations are negative. This indicates that smoking not only has overall effects of increasing body weight in all populations, but also can increase weight in E-A population and decrease weight in C-A, A-A, and H-A populations.

Compared to effects of SNPs not affected by smoking, genetic effects caused by smoking are smaller. The largest effect is 6.94 with heritability 1.74% of rs17094894 near the *VRK1* gene. This shows that effect of smoking on weight-related genes is not so significant as compared to their own effects on body weight. This SNP also has ethnicity-specific additive × dominance epistasis effect with interaction to rs11684785 in the *TMEM163* gene in E-A population ($dae_{1} \overset{\wedge}=$ 4.51 for T/C × C/C and – 4.51 for T/C × T/T), indicating that this SNP is a key factor in the mechanism of weight gain contributed by smoking in all populations, but is also a smoking-related factor in the mechanism of weight gain in E-A population.

SNP rs620175 has many additive-related effects, including ethnicity-specific additive effects in A-A population and three epistasis effects with rs2504934, rs9639575, and rs1277840. The only additive effect due to homozygote genotype A/A of rs1415210 in the *CRB1* gene is highly significant ($a \overset{\wedge}=$ 4.13 for A/A but – 4.13 for T/T, *P*_*EW*_-value < 1 × 10^−22^), and this SNP also has another effect in E-A population ($de_{1} \overset{\wedge}=$ – 4.83). SNP rs1277840 is involved in two effects, one single effect ($de_{3} \overset{\wedge}=$ – 5.11) and one epistasis effect ($dde_{1} \overset{\wedge}=$ 8.34) with interaction to rs620175 in the *SDK1* gene.

### SNPs suppressed by smoking

There were seven highly significant SNPs (*P*_*EW*_-value < 1 × 10^−5^) detected only in the conditional model with 25 genetic effects (Table 3).

SNP rs1467194 could only be detected in the conditional model; whereas SNP rs11684785 could not be detected in this model. However, these two SNPs are located close to each other in the same gene *TMEM163.* The homozygote genotype of rs1467194 takes part in two epistasis effects: additive × additive effect with interaction to rs9639575 ($aa \overset{\wedge}=$ – 3.51 for G/G × T/T and A/A × G/G, and 3.51 for G/G × G/G and T/T × A/A), ethnicity-specific additive × additive effect with rs17094894 in E-A population ($aae_{1} \overset{\wedge}=$ 3.45 for G/G × C/C and A/A × T/T, − 3.45 for G/G × A/A and C/C × T/T), and ethnicity-specific additive × dominance effect with rs17094894 in C-A population ($ade_{2} \overset{\wedge}=$ 8.44 for G/G × C/T but – 8.44 for A/A × C/T).

The dominance × additive effect ($dae_{3} \overset{\wedge}=$ – 11.76 for G/A × T/T but 11.76 for G/A × C/C) of rs2504934 × rs620175 is the negatively largest effect suppressed by smoking with the heritability of 4.42%, which contributes to a large part of the total heritability of epistasis effects in the conditional model. SNP rs2656825 was not detected in the base model, but has highly significant additive effect ($d \overset{\wedge}=$ 9.06, *P*_*EW*_-value < 1 × 10^−64^) in the conditional model. Apart from this single effect, six epistasis effects were detected due to interactions with three SNPs (rs620175, rs1277840 and rs2145965), implying that this SNP is involved in body weight regulation and could be suppressed by smoking.

Smoking also suppresses the ways in which SNPs regulates the body weight via other two important SNPs (rs1415210 and rs2145965). SNP rs1415210 has epistasis effects with rs17094894 ($aa \overset{\wedge}=$ – 6.44 for A/A × C/C and T/T × T/T, 6.44 for A/A × T/T and C/C × T/T; $ade_{4} \overset{\wedge}=$ – 9.97 for A/A × C/T but 9.97 for T/T × C/T). The heterozygote genotype G/C of rs2145965 near the *KIZ* gene has a positive dominance effect ($d \overset{\wedge}=$ 6.42) and three negative effects with interaction to rs2656825 ($ad \overset{\wedge}=$ – 3.17 for A/A × G/C but 3.17 for T/T × G/C, $dd \overset{\wedge}=$ – 7.77, $ade_{1} \overset{\wedge}=$ – 5.17 for A/A × G/C but 5.17 for T/T × G/C).

### Gene ontology analysis

Using ***BioPubInfo*** (https://ibi.zju.edu.cn/biopubinfo/), we also assessed the structural and functional connectivity for the detected candidate genes.

**Figure 2:**
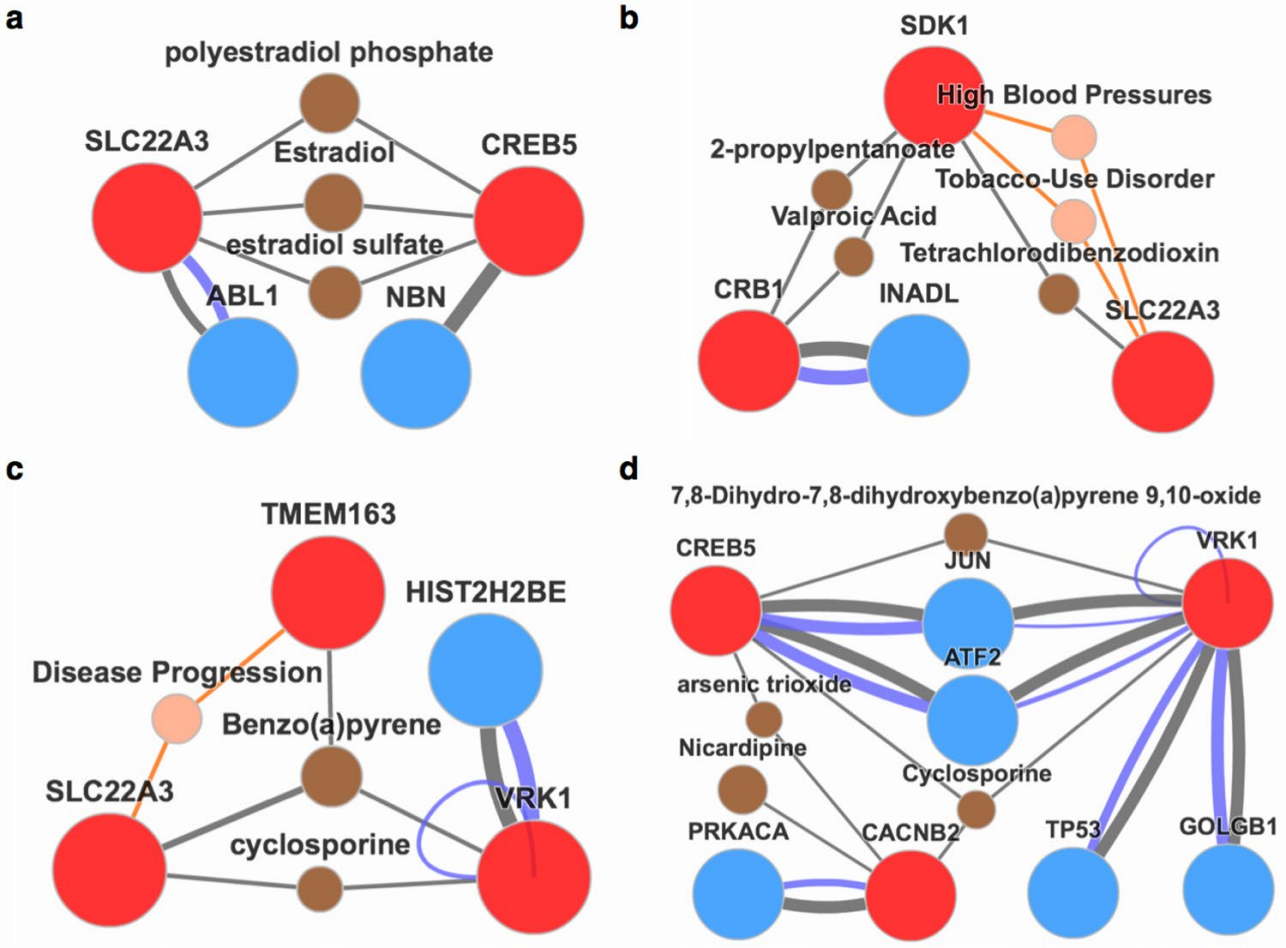
Genetic network of highly significant SNPs for body weight. Red balls = genes detected by our model; Brown balls = related chemicals; Royal blue balls = association genes. Lines = association between genes, diseases, proteins, and chemicals. (**a**) Two SNP-related genes not caused or suppressed by smoking. (**b**) Three SNP-related genes caused by smoking. (**c**) Three SNP-related genes suppressed by smoking. (**d**) Four key related genes that have interesting stories about human weight.

Figure 2a shows the network of two SNP-related genes (*CREB5* and *SLC22A3*) not caused or suppressed by smoking. The *CREB5* gene is connected to the *SLC22A3* gene via three kinds of chemicals (estradiol, polyestradiol phosphate, and estradiol sulfate), which are all related to estrogen. Gonadal hormones can effectively control body weight, the activation of estradiol will affect the body's homeostasis for a long time in female animals, it has similar effects in humans^21^.

Figure 2b shows three SNP-related genes with the largest effects caused by smoking (*CRB1, SDK1, SLC22A3*). The *SDK1* gene is connected to the *CRB1* gene through valproic acid and to the *SLC22A3* gene via tetrachlorodibenzo-p-dioxin (TCDD). These two chemicals were both proved to be related to body weight. A common adverse reaction of valproic acid is weight gain^22^. TCDD is a main compound in cigarettes, and animals treated with TCDD will develop overeating and become obese^23^. SNP rs2504934 in the *SLC22A3* gene has seven kinds of epistasis effects with interaction to rs620175 in *SDK1* gene in all populations (Table 3), indicating that this epistasis is important in body weight regulation, and that these effects may be caused by smoking through TCDD. From gene ontology analysis, the relationship between these three genes became clearer for smoking and body weight.

Figure 2c shows three SNP-related genes (*TMEM163*, *VRK1*, *SLC22A3*) with the largest effects suppressed by smoking. All the three genes are connected to each other by benzopyrene. Long-term exposure to benzopyrene may cause obesity^24^, and overweight/obesity can be experimentally induced by benzopyrene^25^, which is a main compound in cigarettes^26^, and all these three genes have effects contributed or suppressed by smoking. This gene ontology plot proves the reliability of our results in some ways.

Figure 2d shows other three key genes (*CACNB2*, *VRK1*, *CREB5*) that have interesting stories about body weight. The *VRK1* and *CREB5* genes are connected via 7,8-dihydro-7,8-dihydroxybenzo(a)pyrene-9,10-oxide, which is an analogue of benzopyrene. Cyclosporine is connected to genes *CACNB2*, *VRK1* and *CREB5*. Cyclosporine, which is widely used in organ transplantation, was found to cause weight gain after transplantation^27^. The *CREB5* gene and the *VRK1* gene are connected through the *JUN* gene and the *ATF2* gene. CRE (cAMP response element) can bind to the product of the *CREB5* gene with c-Jun (another name of the *JUN* gene). The protein encoded by the ATF2 gene is closely related to CRE and c-Jun. An essay clearly described the regulatory principles and the complex metabolic interaction of CRE, c-Jun, the *ATF2* gene, and the *VRK1* gene^28^. This network may be a way of controlling body weight and the reasons are as follows. CREB (CRE binding protein) and ATF2 could affect the expression of UCP1, a protein essential for the thermogenic function of brown adipose tissue. The brown adipose tissue is related with heat production in human body, and there is a weight loss therapy for the purpose of activating the tissue^29^. Thus, we may have discovered a novel mechanism involving CRE of these four genes that has impacts on body weight and weight-related diseases through a complex metabolic network, the specific reason remained to be revealed.

### Gene effect prediction

There were 15 SNP/epistasis detected in the base model and 22 SNP/epistasis detected in the conditional model. All the 15 SNP/epistasis were also detected in the conditional model. The patterns of gene effects for 15 SNP/epistasis in the conditional model are almost identical with those in the base model (some new effects were detected when setting smoking as a covariant, indicating that these effects are suppressed by smoking). This shows the stability and reasonableness of our conditional method. Although some effects of the 15 SNP/epistasis were not detected in the conditional model (some effects disappeared when setting smoking as a covariant, indicating that these effects are caused by smoking), these effects have little effects on the population and are not large in number. Therefore, we believed that the overall impact of smoking on body weight is produced by suppressing the expression of certain weight-related SNP/epistasis.

**Figure 3:**
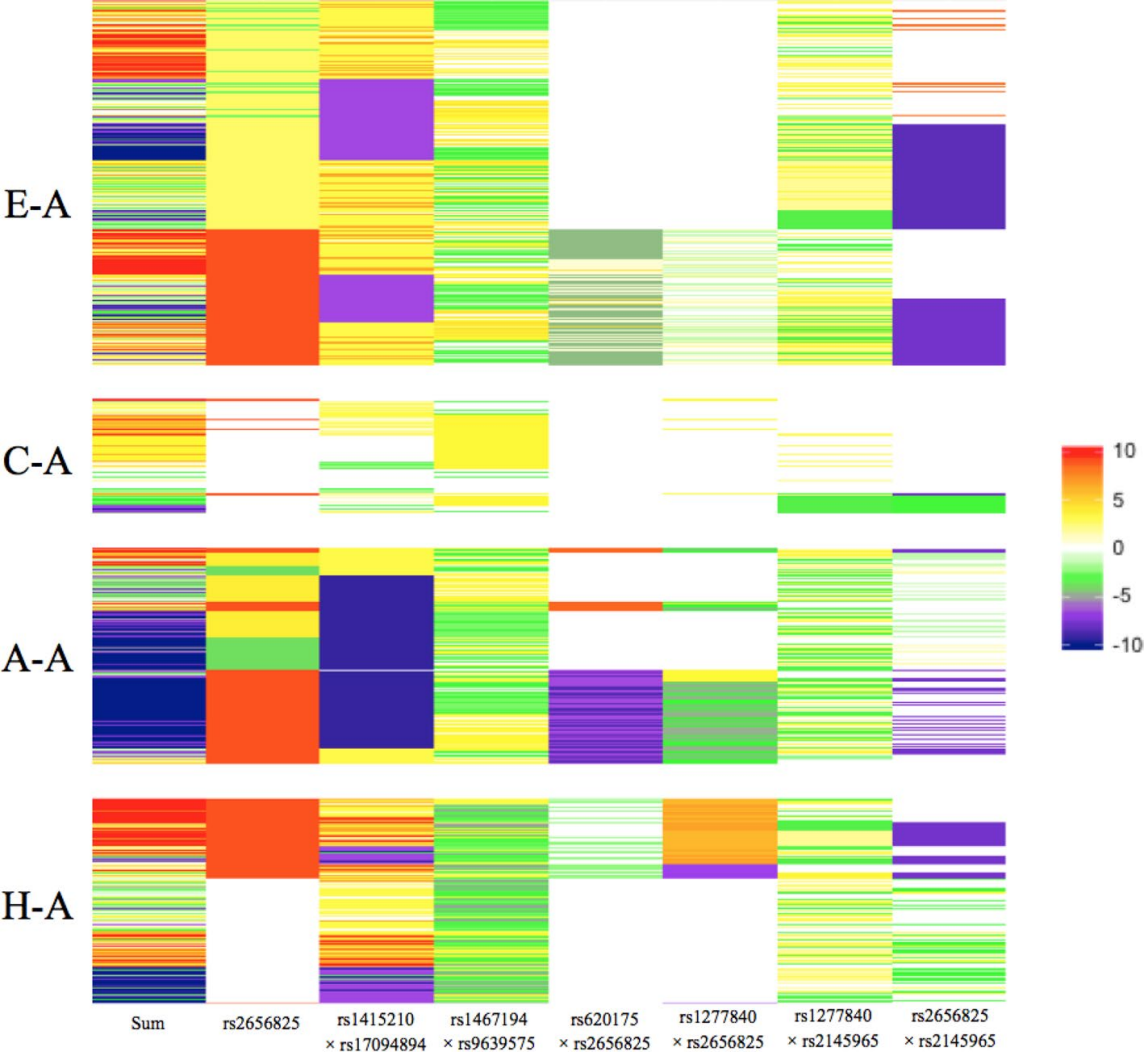
Estimated values of seven SNP/epistasis suppressed by smoking of each individual. Each horizontal line represents an individual, people of same population are present together. From top to bottom, four populations are arranged by European–American (E-A population), Chinese–American (C-A population), African–American (A-A population), and Hispanic American (H-A population). The second to the eighth columns represent effects of SNP/epistasis suppressed by smoking, and the first column is the sum of all columns. The color represents the estimating values of the effects for these SNP/epistasis on specific individual.

In Fig. 3, we present genetic effects of the rest seven SNP/epistasis in the conditional model, which are suppressed by smoking. We arranged individuals of same population together, did clustering on the effects in each population, and then visualized the result by *R* software.

The most important result of Fig. 3 is that smoking has different effects on different populations. The effects of these seven SNP/epistasis were smaller in effect size and less in effects number compared to other three ethnic populations (represented in Fig. 3 as the region of C-A population has large area of blank or light color), showing that smoking has limited effects on C-A population. However, A-A population has large area of dark color, and the color is the darkest in all effects. From the point of single SNP/epistasis, rs620175 × rs2656825 has negative effects (purple color) on some people in A-A population but not in other populations; rs1277840 × rs2656825 has positive effects (orange color) on some people in H-A population but not in other populations. Some other SNP/epistasis have the same phenomenon.

The first column is the sum of all columns, from which we can infer when people quit smoking, he or she will gain or lose how much body weight. In E-A and H-A populations, a small number of people will gain weight heavily (people have red color in the first column), but also a small number of people will lose weight heavily (people have blue color in the first column), and major part of people will have slight weight change. In C-A population, smoking cessation has limited effects on body weight, but in A-A population, a majority of people will lose weight when quitting smoking.

## Discussion

Body weight is a typical complex trait in human that is affected by genetic and environment factors as well as their interactions. In our study, we assessed the genetic effects of individual genes, epistasis effects between pairs of genes, and ethnic-specific interaction effects by mixed linear model approaches. We identified eleven significant SNPs for body weight with *P*_*EW*_-value less than 1 × 10^–5^, and seven of them were found to be within the introns of identified genes. Among all the genetic loci detected, one gene (*CRB1*) has been reported by recent GWAS studies^30^, and three genes (*TMEM163*, *SLC22A3*, *CACNB2*) have been shown to have associations with human body weight.

Gene ontology analysis indicates that there may be new mechanism involving cAMP response element, which may affect body weight. Also, three chemicals (cyclosprine, estradiol, and benzopyrene) have a vast number of associations with genes detected through our gene ontology analysis, which have been reported to be related to body weight.

Smoking behavior as well as body-weight both may have correlative association with some SNPs, but direct biological evidences are insufficient. We have conducted a conditional GWAS to detect the impact of smoking on body weight by comparing the different results between two models (WT and WT|SMK), rather than directly studying the interaction with them. GWAS is hypothesis-free and does not require prior knowledge of a SNP’s biological impact on body weight.

Setting the first few principal components of genetic relationship matrix as covariates in the model is a common method for controlling population stratification in GWAS. The ethnicity-specific effect in our model is not a covariant, but an effect with estimate value, standard error, P-value and heritability. The population stratification due to different ethnic populations can be effectively controlled by ethnicity-specific effect, and there is no need to set principal components in the model as covariates.

Conditional genetic model is an effective method to reveal conditional effects excluding interference factors. When we added smoking variables in the mixed linear model as cofactor, it could reveal net genetic effects after removing influence of smoking. In this way, we can detect SNPs associated with body weight which are affected by smoking. It is worth mentioning that, a SNP will not be detected in the conditional model if it affect body weight only through smoking.

Gene effect prediction shows that smoking has different impacts on different human populations. The genetic effects of smoking cessation on body weight vary from different populations. Body weight of C-A population is not sensitive to smoking, while A-A population has opposite situation. In E-A and H-A populations, people can be divided into several subgroups, and precision medicine and intervention of living habits can be applied on each subgroup for smoking people to keep good fit.

Comparing with low heritability (less than 10%) of weight-related genes estimated by most of the previous GWASs^2,3^, the total heritability of human body weight in this study is up to 61.78% (base model). Majority of the heritability is contribute by ethnicity-specific epistasis $h_{IE}^{2} \overset{\wedge}=$ 26.21% ($h_{AAE}^{2} \overset{\wedge}=$ 1.67%, $h_{ADE}^{2} \overset{\wedge}=$ 13.66%, $h_{DAE}^{2} \overset{\wedge}=$ 5.49%, $h_{DDE}^{2} \overset{\wedge}=$ 5.39%), indicating that epistasis and ethnicity-specific effects are important parts of genetic structure of human body weight.

The heritability of epistasis × ethnicity effects accounts for the largest proportion of total heritability and more than half of the effects are ethnicity-specific effects. The same singular gene could have different impacts on different ethnic groups, therefore ethnicity-specific effects should be considered when designing personalized medicine therapies for weight related diseases.

Over the past decade, dozens of genes associated with body weight have been identified, but biologists have not confirmed most of them. A highly significant gene associated with human body weight detected in our study have been found to be significant in the previous GWAS studies on traits related with body weight. One GWAS assessed the relationship between the *CRB1* gene and human body weight in Wight American^30^. The SNP rs1415210 in the first intron of *CRB1* gene has single dominance effects and also epistasis effects with interaction to many other SNPs (rs7006789, rs2504934, rs17094894), indicating that rs1415210 regulates body weight through different metabolic networks. This gene plays a role in photoreceptor morphogenesis in the retina and may maintain cell polarization and adhesion. However, no evidence has been revealed to support the relationship between this gene and weight.

In this study, we found eight new significant SNPs associated with human body weight. The most important SNP is rs2504934. This SNP not only has the largest positive effect, but also has the largest negative effects that are highly significant ($de_{3} \overset{\wedge}=$ 22.27, *P*_*EW*_-value < 1 × 10^−47^; $de_{4} \overset{\wedge}=$ – 13.67, *P*_*EW*_-value < 1 × 10^−32^), indicating that this SNP is a key factor on body weight regulation. SNP rs2504934 of the first intron of gene *SLC22A3* was detected to be highly significant in both base and conditional models, and has large positive effects. This gene is one of three similar cation transporter genes located in a cluster on chromosome 6, and this cluster was identified as a strong susceptibility locus for coronary artery disease through a genome-wide haplotype association study^31^. Coronary artery disease is one of the obesity-related diseases^32,33^.

SNP rs11684785 and SNP rs1467194 are located in the fourth intron of the *TMEM163* gene. This gene encodes a protein of the same name that is associated with cellular zinc homeostasis^34^. Zinc is of great importance on metabolism, and seriously affects weight and the incidence of type II diabetes^35,36^. In a GWAS of Indian population for type II diabetes, *TMEM163* is the most significant gene associated with type II diabetes^37^.

SNP rs17094894 is near the *VRK1* gene, a gene has been identified to be associated with childhood obesity^38^. SNP rs17094894 has the largest effect with high heritability among effects caused by smoking. In a GWAS that studied the growth of boars, researchers found the association between the *VRK1* gene and weight of boars, and this gene also related to cell growth and division^39^.

SNP rs620175 in the *SDK1* gene has most effects among all SNPs. It has epistasis effects with interactions to other five SNPs, indicating that this gene regulates body weight by lots of mechanisms. Food conversation, which is closely connected to weight growth, is partly contributed by extracellular matrix and cell adhesion protein as SDK1^40^.

SNP rs1277840 is in the fourth intron of the *CACNB2* gene, and most of the effects involving rs1277840 are general effects, indicating that this gene can be expressed stably in all the ethnic groups. This gene is significantly associated with obesity-related diseases involving blood pressure^41,42^. A study on agouti showed that agouti regulates fatty acid synthase and fat storage via calcium channel, which is related to the protein encoded by this gene^43^. This gene was also found in human, and this illustrated that this gene may regulate human body weight via similar ways. In addition, increasing adipocyte intracellular calcium resulted in fat production, and increasing dietary calcium suppressed adipocyte intracellular calcium and thereby modulated energy metabolism and attenuates obesity risk^44^.

Fewer genetic effects were identified in C-A population, compared to the other populations. One possible reason is that the sample population consisted of only 12% Asian Americans. Perhaps a larger sample might help reveal more genetic structure of C-A population. Another problem is that human body weight may be affected by rare alleles, but we used a relatively conservative threshold in the first screen of SNPs to ensure the confidence; some rare alleles with large effects may be filtered out because of low *P-*value. Nonetheless, we still obtained a high total heritability and detected a number of SNPs in four populations with smoking as a covariant. Our results provide new insight in dissecting and understanding the sophisticated genetic architecture of human body weight, which will be useful for designing personalized medicine therapies for complex diseases.

## Data Availability

Raw data is available on request.
